# Post-Exercise Controlled Breathing Enhances Cardiovascular Recovery and Autonomic Balance: A Randomised Crossover Study

**DOI:** 10.3390/medicina62020318

**Published:** 2026-02-03

**Authors:** Eugenijus Trinkunas, Zivile Kairiukstiene, Monika Trinkunaite, Kristina Poderiene, Ruta Brazdzionyte, Jonas Poderys

**Affiliations:** 1 Institute of Sport Science and Innovations, Lithuanian Sports University, 44221 Kaunas, Lithuania; eugenijus.trinkunas@lsu.lt (E.T.); jonas.poderys@lsu.lt (J.P.); 2 Department of Health Promotion and Rehabilitation, Lithuanian Sports University, 44221 Kaunas, Lithuania; kristina.poderiene@lsu.lt (K.P.); ruta.brazdzionyte@stud.lsu.lt (R.B.); 3 Kaunas Hospital, Lithuanian University of Health Sciences, 44307 Kaunas, Lithuania; monika.trinkunaite@kaunoligonine.lt

**Keywords:** hyperventilation, heart rate variability, arterial blood pressure, muscle oxygenation

## Abstract

*Background and Objectives:* Controlled breathing can influence autonomic regulation and haemodynamics; however, the role of its timing relative to exercise remains unclear. *Materials and Methods:* Fourteen healthy, physically active men (mean age 21.8 ± 0.7 years; body mass index within the normal range) participated in this randomised crossover study. Each session consisted of five 5 min cycling bouts at 50% of heart-rate reserve, interspersed with 3 min passive recovery periods. The three conditions were: control (no structured breathing), 30 s hyperventilation (approximately 30 breaths·min^−1^) performed before each bout, and the same hyperventilation performed after each bout. Resting heart rate variability spectra (low-frequency [LF], high-frequency [HF]) were assessed pre- and post-session; arterial blood pressure was measured stage-wise; quadriceps muscle oxygen saturation (StO_2_) was monitored using near-infrared spectroscopy; and a discriminant co-integration index (Dsk) was calculated to integrate multisystem responses. *Results:* Compared with baseline, LF power increased and HF power decreased after exercise in the control and post-exercise hyperventilation conditions (*p* < 0.05), whereas pre-exercise hyperventilation attenuated these shifts. Post-exercise hyperventilation blunted the rise in systolic blood pressure and reduced diastolic blood pressure compared with control (*p* < 0.05). Both breathing interventions accelerated StO_2_ recovery, with higher early recovery StO_2_ following pre-exercise hyperventilation and sustained advantages after post-exercise hyperventilation (moderate-to-extensive effects). Dsk values were consistently highest after exercise, indicating stronger and more coherent multisystem coupling. *Conclusions:* In this acute crossover study of healthy young men, hyperventilation performed before or after exercise induced distinct short-term cardiovascular and muscular responses, reflecting respiratory-driven modulation of haemodynamic and autonomic processes. The timing of hyperventilation influenced these responses, suggesting that deliberate hyperventilation may acutely modify exercise-related regulatory mechanisms.

## 1. Introduction

Regular physical activity (PA) is one of the key factors in maintaining health and preventing cardiovascular disease. Although regular physical activity broadly supports cardiovascular health, aerobic exercise of moderate-to-vigorous intensity is particularly relevant for autonomic and haemodynamic adaptations. Exercise exerts beneficial effects on the cardiovascular system, metabolism, and neural regulation, spanning the entire continuum of physical activity PA levels [1,2,3]. Improving the effectiveness of health-enhancing exercise remains an ongoing challenge, closely related to the diversity of interventions and their interactions [4,5]. A broader arsenal of approaches, including breathing exercises and their individualisation, is increasingly recognised as a means of optimising exercise outcomes, given that breathing is a synergistic process under continuous autonomic regulation [6,7].

Voluntary modulation of breathing frequency and depth can significantly influence vegetative functions and their responses during exercise. 

Controlled breathing, particularly slow and paced breathing patterns, is considered an effective strategy for improving baroreflex sensitivity, enhancing heart rate variability (HRV), and promoting parasympathetic activation [8,9].

Breathing techniques may also attenuate sympathetic dominance by modulating hypothalamic–pituitary–adrenal axis activity, reducing circulating stress hormones such as cortisol, and limiting sympathetic tachycardia. In this way, structured breathing exercises may exert long-term physiologically beneficial effects by helping to maintain balanced autonomic regulation. Consequently, active breathing, breath-holding, and manipulation of the inspiratory-to-expiratory ratio have become important targets of research, although combinations of exercise and breathing tasks have been investigated far less extensively [10,11].

To evaluate the effects of breathing exercises on the cardiovascular system, it is essential to examine several interrelated indices: HRV, arterial blood pressure (ABP), and skeletal muscle StO_2_. These markers reflect different yet tightly linked physiological mechanisms, including autonomic regulation, haemodynamics, and tissue metabolism [7,12,13]. HRV is a sensitive biomarker of autonomic function, providing insight into the balance between sympathetic and parasympathetic influences. Higher HRV is associated with better adaptation to exercise and stress, whereas reduced HRV has been linked to autonomic dysregulation and increased cardiovascular risk. Breathing exercises, particularly slow diaphragmatic breathing, can directly influence vagal activity and may increase HRV [14,15]. Importantly, these autonomic benefits have been consistently reported for slow and controlled breathing and should not be directly extrapolated to rapid or deep breathing patterns such as hyperventilation, which induce distinct physiological responses related to hypocapnia and respiratory alkalosis [16].

ABP exhibits the so-called post-exercise hypotension phenomenon, a transient reduction in blood pressure associated with vasodilation and increased peripheral blood flow.

Breathing interventions may influence post-exercise blood pressure responses; however, such effects are strongly dependent on the specific breathing pattern applied and associated changes in arterial CO_2_ levels [17,18].

StO_2_ is directly related to tissue oxygenation and metabolic processes. During exercise, StO_2_ decreases as a result of increased oxygen utilisation during mitochondrial oxidative phosphorylation, a normal physiological response [19]. In the recovery phase, the rate of StO_2_ reoxygenation is a key indicator of peripheral blood flow and metabolic recovery. Certain breathing interventions may influence StO_2_ recovery after exercise through altered haemodynamics and peripheral oxygen balance; however, StO_2_ primarily reflects the balance between oxygen delivery and utilisation and does not provide a direct measure of mitochondrial activity or ventilation–perfusion matching [16].

Given that breathing interventions can modulate autonomic nervous system activity, support HRV, and influence blood pressure recovery and muscle oxygen dynamics after exercise [20,21,22], it is relevant to clarify how the timing and nature of such interventions interact with acute exercise responses. In this context, hyperventilation should be regarded as an acute physiological intervention rather than a therapeutic or health-promoting breathing strategy, as it induces hypocapnia and respiratory alkalosis that may substantially alter autonomic, haemodynamic, and cerebrovascular responses. Therefore, this study examined three cycling protocols with and without hyperventilation tasks performed either before or after each exercise bout.

The aim was to characterise the acute physiological effects of hyperventilation in relation to exercise and to clarify its short-term influence on cardiovascular responses and recovery processes, without extrapolation to long-term adaptations or health benefits.

## 2. Materials and Methods

### 2.1. Trial Design

**Figure 1:**
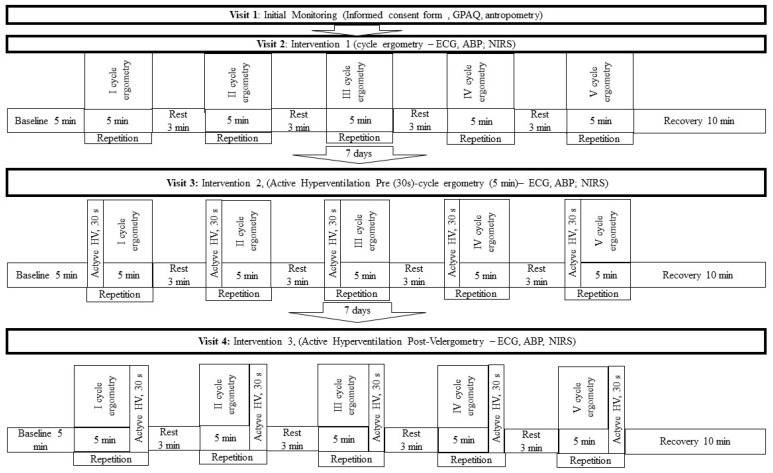
Overview of the study protocols across the three intervention conditions. GPAQ: Global Physical Activity Questionnaire; ECG: electrocardiogram; ABP: arterial blood pressure; NIRS: near-infrared spectroscopy; HV: hyperventilation; min: minutes; s: seconds.

This study was conducted as a randomised crossover experimental trial. Before the initial measurements, a meeting was held during which participants were briefed on the study protocol and instructed to refrain from eating for 3 h prior to the examination. All assessments were conducted using the same procedures and under consistent conditions. The study included three types of intervention in a randomised crossover design: a control intervention, in which participants performed a cycling protocol with 3 min rest intervals; an intervention with hyperventilation performed before each exercise bout; and an intervention with hyperventilation performed immediately after each exercise bout during the recovery phase. A 7-day washout period separated each visit (Figure 1). Hyperventilation was implemented as an acute physiological task intended to transiently modify respiratory and cardiovascular responses, and was not designed or presented as a therapeutic or health-promoting intervention.

Each protocol consisted of five 5 min cycling bouts at 50% of heart rate reserve, interspersed with 3 min passive recovery periods, performed on a Monark 928 G3 ergometer (Monark Exercise AB, Vansbro, Sweden). Exercise intensity was individualised using the Karvonen formula [23] and maintained with continuous electrocardiogram (ECG) monitoring and workload adjustments (typically 95–140 W). A standardised 5 min warm-up at 1 W/kg preceded each session. Hyperventilation tasks, when applied, were performed as described below.

The procedures consisted of standardized physical exercise and voluntary breathing tasks commonly used in exercise physiology research; no drugs, medical devices, invasive procedures, or clinical treatments were administered, and no clinical outcomes were assessed. Participation was permitted following medical clearance by a physician. The Lithuanian Bioethics Committee approved the study (Authorisation for Biomedical Research, 23 January 2020, No. L-20-1/1), and all procedures were carried out in accordance with the ethical guidelines of the Declaration of Helsinki.

### 2.2. Participants

The study involved 14 physically active, healthy, non-smoking men (age: 21.8 ± 0.7 years; height: 183.0 ± 1.3 cm; body mass: 77.9 ± 1.3 kg). All participants had normal resting blood pressure and body mass index, reported no known health problems, and were not taking any medications or nutritional supplements. Based on the Global Physical Activity Questionnaire, participants were classified as having a moderate level of regular PA. All individuals had been training at a gym for a minimum of 1 year and reported performing at least 3 h of weekly exercise, including both aerobic and resistance training. Prior to participation, all subjects received medical clearance, were informed about the purpose and procedures of the study, and provided written informed consent.

### 2.3. Physical Interventions and Breathing Protocol

This study examined the acute effects of three intervention protocols combining endurance-type cycling with or without a standardised hyperventilation task applied as an acute physiological stimulus. In the control protocol (intervention 1), cycling was performed without any additional breathing tasks. In intervention 2, participants performed a standardised hyperventilation task immediately before each 5 min cycling bout. In intervention 3, the same hyperventilation task was performed immediately after each 5 min cycling bout, during the subsequent 3 min passive recovery period.

The hyperventilation task consisted of 30 s of active, deep breathing at a target rate of 30 breaths per minute, corresponding to approximately 15 inhalation–exhalation cycles. Participants were instructed to breathe through the mouth using deep, vigorous inspirations and expirations, guided by a metronome to maintain the target frequency. Before the first experimental visit, participants were familiarised with the breathing pattern to ensure consistent performance across sessions. Throughout each breathing bout, the investigator directly observed participants to monitor their well-being and any possible symptoms of hypocapnia (e.g., dizziness, tingling, visual disturbances). Participants were explicitly advised to stop the breathing task and inform the investigator if such symptoms occurred; none were reported.

Following completion of the five exercise–recovery cycles, all participants remained seated on the ergometer for an additional 10 min recovery period to assess the return of cardiovascular and muscular indices towards baseline.

Respiratory gas exchange variables, including arterial or end-tidal CO_2_, were not measured during the hyperventilation task; therefore, the magnitude of hypocapnia can only be inferred indirectly based on the breathing protocol.

### 2.4. Assessments

#### 2.4.1. Measures of PA

PA was assessed using the Global Physical Activity Questionnaire (GPAQ), a widely validated and reliable tool developed by the World Health Organization [24]. The GPAQ captures PA across three domains: occupational activity, active transportation, and leisure-time or recreational activity. It consists of 16 questions assessing the intensity, frequency, and duration of PA. In the present study, all participants reported engaging in at least moderate-intensity PA.

#### 2.4.2. Body Composition

Participants’ height and body mass were assessed using calibrated instruments, with measurements taken while subjects were barefoot and wearing only underwear. Stature was measured with a SECA^®^ 213 stadiometer (SECA GmbH & Co., Hamburg, Germany), and body mass was assessed using a TANITA^®^ BC-545 scale (TANITA Corporation, Tokyo, Japan).

#### 2.4.3. Oxygen Saturation

Changes in StO_2_ during the protocols were assessed using non-invasive near-infrared spectroscopy with a photosensor device (Hutchinson Technology, Hutchinson, MN, USA). The sensor was attached to the main working muscle group, the quadriceps (vastus lateralis). StO_2_ levels were continuously recorded throughout the entire protocol, including rest before the interventions, warm-up, exercise, and recovery phases, as well as during the 10 min post-exercise recovery period following the final workload stage. For analysis, all values were normalised to baseline and expressed as percentages. Near-infrared spectroscopy provides an index of relative skeletal muscle oxygen saturation reflecting the balance between oxygen delivery and utilisation; it does not directly assess mitochondrial oxidative phosphorylation, ventilation–perfusion matching, or lactate metabolism.

#### 2.4.4. ABP Measurements

ABP was measured using a cuff-based auscultatory method, applying Korotkoff sounds to identify systolic blood pressure (SBP) and diastolic blood pressure (DBP) (Riester, Jungingen, Germany). Measurements were taken in the seated position on the same arm in all sessions, using an appropriately sized cuff. Resting arterial blood pressure was measured after several minutes of quiet seated rest prior to the intervention. ABP was assessed at rest before the interventions, immediately after each cycling stage, and during the post-exercise recovery phase at 3, 5, and 10 min. Arterial blood pressure was analysed as a haemodynamic outcome variable, without assuming direct correspondence with autonomic nervous system activity.

#### 2.4.5. ECG Measurements

A standard 12-lead ECG was recorded using the CardioScout Multi ECG system (Medset, Hamburg, Germany). Recordings were performed continuously at baseline, throughout the cycling exercise, and during the 10 min seated recovery phase. After electrode placement, participants rested quietly to establish a stable baseline, followed by an additional 1 min seated rest period on the ergometer. In all three research protocols, participants cycled at 50% heart rate reserve, with workload continuously adjusted according to real-time heart rate feedback to maintain the target zone. HRV spectral indices in the low-frequency (LF) and high-frequency (HF) bands were derived from ECG recordings obtained at rest before and after the interventions. LF and HF spectral components were analysed as indirect markers of autonomic modulation. No one-to-one interpretation of LF as sympathetic or HF as parasympathetic activity was assumed. Discriminant values were calculated from HRV data as described below, incorporating both amplitude parameters (time-domain and spectral power) and frequency-domain parameters (LF and HF components).

### 2.5. Data Analysis

#### 2.5.1. Statistical Analysis

All statistical analyses were performed using IBM SPSS Statistics version 26.0 (IBM Corp., Armonk, NY, USA). Data are reported as mean ± standard deviation, and error bars in the figures represent the standard deviation. The normality of distributions was assessed using the Shapiro–Wilk test (n = 14). For repeated-measures analyses, sphericity was tested with Mauchly’s test; when violated, Greenhouse–Geisser corrections (or Huynh–Feldt when ε > 0.75) were applied, and corrected *p*-values are reported. Because all participants completed all three interventions, both factors were treated as within-subjects: Time (e.g., pre vs. post, or rest, warm-up, exercise, recovery) and Protocol (intervention 1—control; intervention 2—hyperventilation before exercise; intervention 3—hyperventilation after exercise). Two-way repeated-measures analysis of variance with both factors as within-subjects was used to test main effects and interactions. When significant effects were detected, post hoc pairwise comparisons with Holm–Bonferroni correction were conducted. Effect sizes were expressed as Cohen’s d for pairwise comparisons and partial eta squared (η^2^p) for analysis of variance. Effect size thresholds were interpreted according to contemporary methodological guidelines, with values of 0.1, 0.4, and 0.8 corresponding to small, moderate, and large effects, respectively [25]. In addition, 95% confidence intervals were calculated for effect size estimates to support interpretation of the magnitude and precision of observed effects [26]. Qualitative descriptors (small, moderate, large) were used only when supported by these numerical indices. Statistical significance was set at *p* < 0.05.

Effect sizes were reported to describe the magnitude of observed differences; however, conclusions regarding physiological relevance were drawn primarily from statistically significant results.

#### 2.5.2. Mathematical Methods

Conventional statistical methods based solely on average values cannot fully capture the complex interactions between physiological systems during exercise. To address this limitation, an algebraic co-integration approach was used to analyse how two physiological signals co-vary over time [27]. The signals were recorded at discrete time points and normalised to the [0; 1] range, ensuring comparability by scaling values to a common interval. At baseline, values were further standardised to 100%.

From these normalised data, two primary parameters were computed: the difference (dfrA_n_ = x_n_ − y_n_) between paired observations x_n_ and y_n_, and a co-diagonal product (cdpA_n_), which reflects how changes at neighbouring time points interact. These two components were combined into a discriminant index, Dsk, defined as DskA_n_ = (dfrA_n_)^2^ + 4·cdpA_n_. In this framework, smaller Dsk values (approaching zero) indicate stronger physiological coupling between the two signals, whereas larger Dsk values indicate weaker coupling and greater divergence of their dynamics. In the present study, discriminant values were calculated for selected pairs of cardiovascular indices to provide an integrated measure of their interaction during exercise and recovery.

## 3. Results

**Figure 2:**
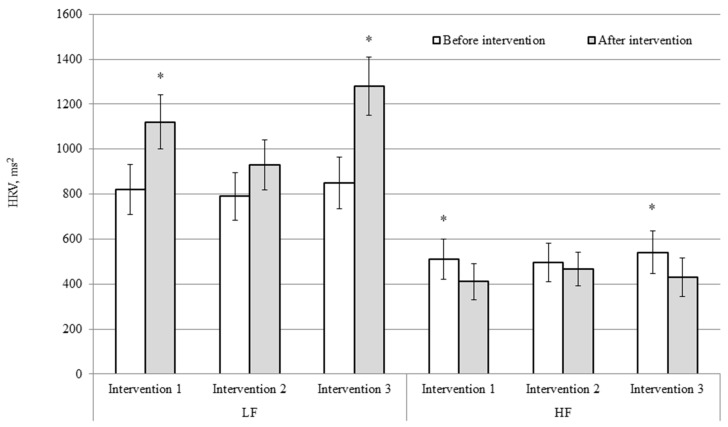
Changes in HRV spectral indices before and after the interventions across the three intervention protocols (intervention 1, intervention 2, intervention 3). HRV: heart rate variability; LF: low frequency; HF: high frequency. Data are presented as mean ± SEM; * *p* < 0.05.

HRV spectral indices were recorded at rest before and after the interventions (Figure 2). At baseline, LF power was similar across the three protocols, with mean values of 820 ± 110 ms^2^ in intervention 1, 790 ± 105 ms^2^ in intervention 2, and 850 ± 115 ms^2^ in intervention 3. Following the interventions, LF power increased in intervention 1 (to 1120 ± 120 ms^2^, *p* < 0.05) and intervention 3 (to 1280 ± 130 ms^2^, *p* < 0.05), while a more modest, non-significant increase was observed in intervention 2 (to 930 ± 110 ms^2^, ns). By contrast, HF power showed a decreasing trend after exercise. At baseline, HF power averaged 510 ± 90 ms^2^ in intervention 1, 495 ± 85 ms^2^ in intervention 2, and 540 ± 95 ms^2^ in intervention 3. After the interventions, HF power decreased significantly in intervention 1 (to 410 ± 80 ms^2^, *p* < 0.05) and intervention 3 (to 430 ± 85 ms^2^, *p* < 0.05), whereas in intervention 2 the reduction was small and non-significant (to 465 ± 75 ms^2^, ns). Overall, these results indicate that interventions 1 and 3 were associated with higher LF and lower HF absolute power at rest after exercise, reflecting altered autonomic modulation following the exercise bouts. By contrast, hyperventilation applied before exercise (intervention 2) was associated with smaller changes in LF and HF power following exercise.

For the hyperventilation-after-exercise intervention, LF power increased significantly from pre- to post-exercise (t(7) = 2.71, *p* = 0.030), corresponding to a large effect size (d = 1.10, 95% CI [0.43, 1.77]). HF power showed a non-significant decrease (t(7) = −1.69, *p* = 0.135), with a moderate effect size (d = 0.80, 95% CI [0.19, 1.41]). Overall, this intervention was associated with pronounced changes in HRV spectral components following exercise. In contrast, hyperventilation performed before exercise (intervention 2) resulted in small, non-significant effect sizes (d ≤ 0.35), indicating relatively minor changes in HRV parameters.

**Figure 3:**
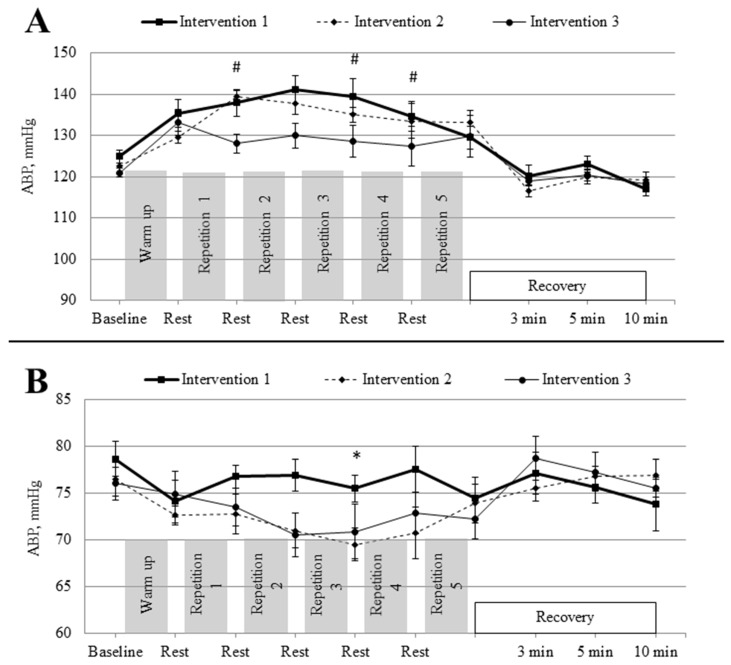
Changes in (**A**) systolic and (**B**) diastolic blood pressure during rest, warm-up, and workload stages across three research protocols (intervention 1, intervention 2, intervention 3). Data are presented as mean ± SEM. * *p* < 0.05 indicates a significant difference for intervention 1 vs. interventions 2 and 3; ^#^ *p* < 0.05 indicates a significant difference for intervention 3 vs. interventions 1 and 2.

Figure 3 illustrates the dynamics of ABP across the three intervention protocols. In intervention 1, resting SBP was 124.9 ± 6.2 mmHg. Following the first workload stage, SBP increased to 137.9 ± 13.2 mmHg (*p* < 0.05), reached 141.1 ± 13.4 mmHg after the second stage (*p* < 0.05), and 139.5 ± 16.7 mmHg after the third stage (*p* < 0.05), changes corresponding to moderate-to-large effects. In intervention 2, resting SBP was 124.4 ± 7.8 mmHg. After warm-up, SBP rose to 131.3 ± 9.0 mmHg (*p* < 0.05), then to 139.4 ± 6.7 mmHg after the first workload stage (*p* < 0.001); it remained at 139.5 ± 7.2 mmHg after the second stage (*p* < 0.01), indicating large-to-very-large effects. In intervention 3, resting SBP was 121.5 ± 3.4 mmHg. After warm-up, values increased to 133.6 ± 6.7 mmHg (*p* < 0.05), and following the second workload stage reached 136.5 ± 9.1 mmHg (*p* < 0.01), reflecting moderate-to-large effects.

Between-protocol comparisons revealed significant differences. After the first workload stage, SBP was significantly lower in intervention 3 than in intervention 1 (123.5 ± 3.2 vs. 137.9 ± 4.8 mmHg; *p* < 0.01) and intervention 2 (123.5 ± 3.2 vs. 139.4 ± 2.4 mmHg; *p* < 0.001), reflecting large-to-very-large effect sizes and highlighting a pronounced difference in SBP responses between post-exercise hyperventilation and the other protocols.

With respect to DBP, resting DBP in the control protocol (intervention 1) was 78.6 ± 2.7 mmHg. In intervention 3, resting DBP was significantly lower than in the control (71.2 ± 2.6 vs. 78.6 ± 2.7 mmHg; *p* < 0.05), indicating a moderate-to-large effect. In intervention 2, resting DBP was 75.5 ± 2.8 mmHg. After the third workload stage, DBP decreased to 69.5 ± 2.6 mmHg (*p* < 0.05), indicating a moderate negative effect. When comparing protocols, DBP during the third workload stage was significantly lower in intervention 2 compared with the control (69.5 ± 2.6 vs. 75.5 ± 2.1 mmHg; *p* < 0.01), representing a large effect.

**Figure 4:**
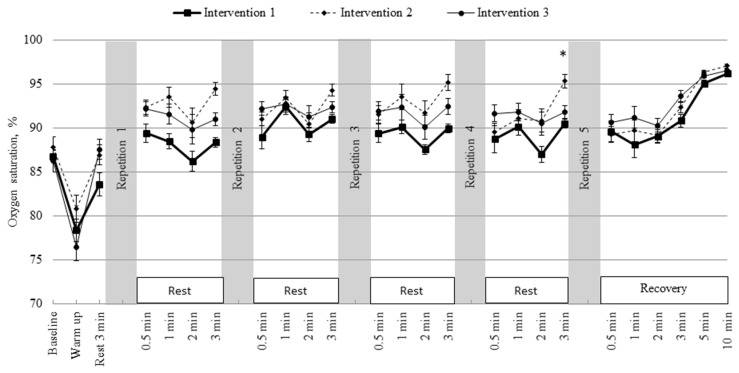
Changes in quadriceps muscle oxygen saturation during baseline, warm-up, interventions, and recovery across three intervention protocols. Data are presented as mean ± SEM. * *p* < 0.05 indicates a significant difference for intervention 2 vs. interventions 1 and 3.

Figure 4 shows the dynamics of quadriceps muscle StO_2_ across the three intervention protocols. At rest, StO_2_ values did not differ significantly and were 86.8% ± 1.3% in intervention 1, 87.8% ± 0.8% in intervention 2, and 86.2% ± 1.2% in intervention 3. Following warm-up, StO_2_ decreased in all protocols, with the largest reduction in intervention 3 (76.5% ± 1.6%) compared with intervention 1 (78.4% ± 1.6%) and intervention 2 (80.8% ± 1.3%). After 3 min of rest, StO_2_ increased to 83.6% ± 2.1% in intervention 1, 86.9% ± 1.3% in intervention 2, and 87.6% ± 1.1% in intervention 3.

During subsequent resting phases after each intervention stage, StO_2_ progressively increased across all protocols, with more pronounced changes in interventions 2 and 3, where breathing tasks were applied. At the first intervention stage, StO_2_ reached 89.4% ± 1.5% in intervention 1, 92.3% ± 1.0% in intervention 2, and 92.2% ± 0.8% in intervention 3. At the first recovery minute, saturation was highest in intervention 2 (93.5% ± 0.8%) and lowest in intervention 1 (88.5% ± 1.9%), with a very large effect size between these protocols. This pattern persisted at later stages: following the third intervention, at the 2 min mark, StO_2_ was 87.6% ± 1.8% in intervention 1, 91.7% ± 0.5% in intervention 2, and 90.1% ± 1.4% in intervention 3.

During the final recovery period after all interventions, StO_2_ gradually increased in all protocols. After 1 min, values were 88.1% ± 1.6% in intervention 1, 89.7% ± 1.5% in intervention 2, and 91.2% ± 1.2% in intervention 3. Although these differences were not statistically significant, a large effect size was observed between interventions 1 and 3. After 3 min, values were 90.8% ± 1.1%, 92.3% ± 0.4%, and 93.6% ± 0.7%, respectively, again showing a large effect size between interventions 1 and 3. At 5 min, StO_2_ reached 95.1% ± 0.6% in intervention 1, 96.4% ± 0.4% in intervention 2, and 95.9% ± 0.2% in intervention 3, and after 10 min, 96.2% ± 0.4%, 97.0% ± 0.2%, and 96.5% ± 0.2%, respectively, with effect sizes indicating moderate-to-large differences between protocols despite the absence of statistically significant group effects.

**Figure 5:**
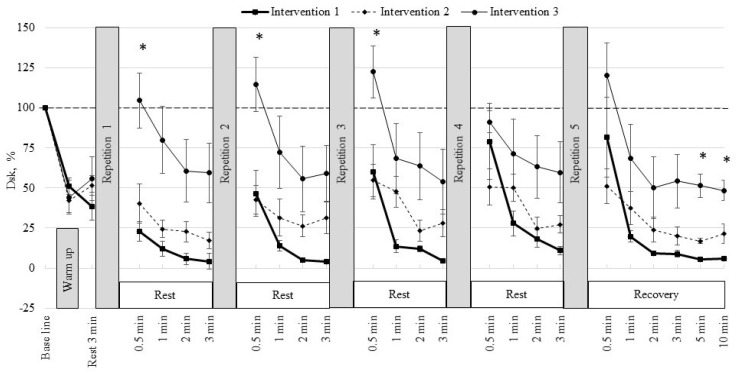
Changes in discriminant values during baseline, warm-up, interventions, and recovery across three intervention protocols. Data are presented as mean ± SEM. * *p* < 0.05 indicates a significant difference for intervention 3 vs. interventions 1 and 2.

Figure 5 presents the dynamics of discriminant values across the three interventions. At baseline, all values were normalised to 100%. After warm-up, discriminant values decreased in all protocols, reaching 51.3% ± 5.2% in intervention 1, 42.1% ± 7.4% in intervention 2, and 44.3% ± 10.7% in intervention 3. After 3 min of rest, values increased to 38.6% ± 8.8%, 51.5% ± 6.1%, and 55.8% ± 13.5%, respectively, but remained below baseline. During the first intervention stage, values dropped to 22.7% ± 6.2% in intervention 1 and 40.4% ± 12.3% in intervention 2, whereas in intervention 3 they increased to 104.6% ± 17.1%, with significant differences between interventions 1 and 2 compared with intervention 3 (*p* < 0.05). At 1–3 min of recovery, values remained lowest in intervention 1 and highest in intervention 3, with large-to-very-large effect sizes.

A similar pattern was observed during subsequent intervention stages. After intervention 3, discriminant values reached 60.0% ± 17.0%, 54.7% ± 10.2%, and 122.7% ± 16.3% in interventions 1, 2, and 3, respectively, with follow-up measurements confirming very large effects between interventions 1 and 3 and moderate-to-large effects between interventions 2 and 3 (*p* < 0.05). During the fifth intervention, values were 81.6% ± 25.1%, 50.9% ± 10.9%, and 120.2% ± 20.6% after 30 s in interventions 1, 2, and 3, respectively, with consistent differences across recovery time points. In the final recovery phase, discriminant values gradually declined, but remained lowest in intervention 1 and highest in intervention 3. After 5 min, they were 5.3% ± 0.2%, 16.9% ± 1.7%, and 51.4% ± 7.4%, and after 10 min 5.7% ± 0.6%, 21.4% ± 6.2%, and 48.3% ± 6.3%, respectively. Differences between interventions 1 and 2, compared with intervention 3, remained significant (*p* < 0.05), indicating very large and moderate effects.

## 4. Discussion

In the context of health promotion, assessing cardiovascular functional indices is essential for understanding the body’s response to exercise and for designing evidence-based interventions. While exercise tests and cardiovascular responses have been extensively described in the literature, the combined effects of structured breathing tasks and exercise have received less attention [28,29,30]. The present study sought to address this gap by examining the acute physiological responses to three intervention protocols—control, hyperventilation before exercise, and hyperventilation after exercise—on autonomic indices, arterial blood pressure dynamics, skeletal muscle oxygen saturation, and an integrated discriminant index.

Spectral HRV analysis showed that in the control condition and in the protocol with post-exercise hyperventilation, LF power increased, while HF power decreased at rest after the cycling bouts. These changes are consistent with the well-documented acute autonomic adjustments following dynamic exercise, reflecting altered cardiac autonomic modulation during early recovery [31]. HRV spectral components were analysed using absolute LF and HF power values. Normalised units were not applied, as the study focused on within-condition acute responses rather than inter-individual comparisons. Moreover, as breathing frequency was not controlled during post-intervention measurements, particularly following hyperventilation, LF and HF power values may partly reflect respiratory-driven modulation of heart rate variability. Therefore, these findings should be interpreted cautiously and descriptively rather than as precise indicators of autonomic balance. Such changes support increased heart rate, myocardial contractility, and peripheral vascular tone during and immediately after exercise. It is important to emphasise that LF and HF components represent indirect and composite markers of autonomic regulation, and their interpretation as pure indices of sympathetic or parasympathetic activity has recognised limitations [32]. In intervention 2, no significant changes in LF or HF power were observed at rest following exercise. This absence of marked HRV alterations seems to be rather a neutral or potentially compensatory response occurring in the context of acute respiratory manipulation combined with exercise-induced physiological stress [33].

The three interventions elicited distinct cardiovascular responses during workload. In the control condition (intervention 1), systolic blood pressure increased progressively with workload, reflecting the typical haemodynamic response to dynamic exercise [34], while diastolic blood pressure remained relatively stable. In intervention 2, pre-exercise hyperventilation was associated with higher systolic blood pressure during exercise. This response likely reflects altered haemodynamic regulation associated with hypocapnia rather than improved autonomic or baroreflex control, and it did not prevent the exercise-induced increase in blood pressure [35]. In intervention 3, lower systolic and diastolic blood pressure values were observed during recovery. These changes should be interpreted cautiously, as reductions in blood pressure following hyperventilation may arise from hypocapnia-related alterations in vascular tone, cerebral and peripheral vasoconstriction, and changes in venous return rather than improved autonomic regulation or endothelial function [36].

Across all three interventions, skeletal muscle oxygen saturation displayed the expected pattern of a marked decrease during exercise followed by recovery toward baseline during the post-exercise period. Across all three interventions, skeletal muscle StO_2_ displayed the expected pattern of a marked decrease during exercise followed by recovery toward baseline during the post-exercise period. These changes reflect dynamic adjustments in the balance between oxygen delivery and utilisation within the microcirculation during exercise and recovery [37]. In interventions incorporating breathing tasks, StO_2_ recovery occurred more rapidly. Post-exercise hyperventilation was implemented as a rapid, deep breathing task rather than a slow, controlled breathing exercise typically associated with beneficial autonomic effects in the literature [38]. StO_2_ does not provide a direct measure of mitochondrial activity, muscle perfusion, or ventilation–perfusion matching, and is strongly influenced by haemodynamic factors, blood volume shifts, and microcirculatory redistribution. Accordingly, faster reoxygenation should be interpreted as a descriptive physiological response rather than evidence of enhanced metabolic clearance, improved muscle perfusion, or superior recovery capacity [39]. Moreover, the intervention should not be interpreted as having health-promoting autonomic effects, which are normally observed with slow, shallow breathing protocols.

Discriminant analysis provided an integrative mathematical description of multisystem responses under different intervention conditions by quantifying changes in the dynamic relationship between selected physiological indicators. Within the framework of the algebraic co-integration method, lower Dsk values indicate a stronger coupling between the evaluated signals, reflecting more synchronous behaviour of the underlying physiological mechanisms, whereas higher Dsk values indicate a weakening or reorganisation of these relationships [27]. In the present study, higher discriminant values observed in intervention 3 should not be interpreted as evidence of enhanced autonomic regulation or coordinated physiological adaptation. Unlike training studies in which sustained decreases in discriminant values over time have been associated with long-term systemic adaptation and optimisation of recovery processes, the observed increases in Dsk in this acute protocol most likely reflect condition-specific alterations in system dynamics induced by respiratory manipulation during early recovery. Such changes may indicate faster or differently organised recovery processes. Dsk should be interpreted as a descriptive indicator of how the interaction between cardiovascular and peripheral signals changes under specific experimental conditions, rather than as a marker of functional benefit. In this context, discriminant analysis serves as a sensitive mathematical tool for capturing subtle, condition-dependent variability in complex dynamic systems during exercise and recovery, complementing conventional analyses based on isolated physiological variable

Several limitations should be acknowledged. The sample consisted of a small and homogeneous group of healthy, physically active young men, limiting generalisability to other populations. An a priori power analysis was not performed, as the study was exploratory in nature; however, the crossover design increased statistical efficiency by minimising between-subject variability. In addition, direct measurements of respiratory gases, such as end-tidal or arterial CO_2_, were not obtained; therefore, the magnitude of hypocapnia and respiratory alkalosis can only be inferred indirectly. Although the endurance-type cycling protocol used in this study enabled controlled assessment of repeated exercise–recovery cycles, it does not provide the comprehensive physiological information obtainable from full cardiopulmonary exercise testing (CPET) [40,41]. Finally, the study focused exclusively on acute physiological responses and did not assess exercise tolerance, fatigue development, or long-term adaptations.

## 5. Conclusions

In this acute crossover study of healthy young men, hyperventilation performed before or after exercise induced distinct short-term changes in cardiovascular and muscular physiological indices, reflecting acute respiratory-driven modulation of haemodynamic and autonomic processes. The timing of hyperventilation influenced the pattern of these responses, indicating that hyperventilation represents a potent physiological stimulus capable of modifying exercise-related regulatory mechanisms. While the present findings are limited to acute effects, they suggest that hyperventilation may have relevance within training or conditioning contexts when applied deliberately and interpreted within its physiological framework.

## Data Availability

The datasets generated and analysed during the study are available from the corresponding author upon reasonable request.
